# 2314. Hospitalizations and Antibiotic Use in the Year Prior to an Incident C. difficile Infection for Medicare Beneficiaries in Four States, 2016–2018

**DOI:** 10.1093/ofid/ofac492.146

**Published:** 2022-12-15

**Authors:** Kelly M Hatfield, James Baggs, Sujan Reddy, Rasaki Aranmolate, James Meek, Scott Fridkin, Jill Szydlowski, Trupti T Hatwar, Ghinwa Dumyati, Jasmine Watkins, Christopher Wilson, L Clifford McDonald, John A Jernigan, Alice Guh

**Affiliations:** Centers for Disease Control and Prevention, Atlanta, Georgia; Centers for Disease Control and Prevention, Atlanta, Georgia; CDC, Atlanta, GA; Centers for Disease Control and Prevention, Atlanta, Georgia; Connecticut Emerging Infections Program, New Haven, Connecticut; Emory University School of Medicine, Atlanta, GA; University of Rochester Medical Center, Pittsford, New York; University of Rochester Medical Center, Pittsford, New York; University of Rochester Medical Center, Pittsford, New York; Tennessee Department of Health, Nashville, Tennessee; Tennessee Department of Health, Nashville, Tennessee; Centers for Disease Control and Prevention, Atlanta, Georgia; CDC, Atlanta, GA; CDC, Atlanta, GA

## Abstract

**Background:**

Studies describing risk factors for *Clostridioides difficile* infection (CDI) are often limited in their ability to identify potentially important exposures occurring long before diagnosis. We describe hospitalizations and antibiotic use (AU) occurring up to one year prior to CDI diagnosis among Medicare beneficiaries.

**Methods:**

We studied incident CDI cases (positive *C. difficile* test in a person ≥65 years without a positive test in the prior 8 weeks) identified during 2016–2018 through population-based CDI surveillance from four states participating in the Centers for Disease Control and Prevention’s Emerging Infections Program. The analysis included specimens collected in all settings and was limited to case patients who were identified as having fee-for-service Medicare and Part D drug coverage for the year preceding specimen collection. Inpatient hospitalization data was extracted from Medicare Provider Analysis and Review (MEDPAR) files and outpatient AU (prescriptions filled) was determined using Part D drug event files. Timing of hospitalizations and antibiotic prescriptions were described as recent (0–3 months prior to specimen collection) or remote (4–12 months prior).

**Results:**

Of 1,953 CDI cases, 1,594 (82%) filled ≥1 course of outpatient antibiotics in the prior year; 805 (41%) filled an antibiotic both recently and remotely, 497 (25%) only remotely, and 292 (15%) only recently.

Cases with outpatient AU received a median of 23.5 (IQR 12–46) total days supplied, and a median of 2 different antibiotic classes (IQR 1 – 3). The most frequent antibiotic classes filled include fluoroquinolones (17% of all antibiotics filled), 1^st^ generation cephalosporins (10%), and folate pathway inhibitors (10%).

Overall, 1,314 (67%) cases were hospitalized in the prior year; 569 (29%) were hospitalized both recently and remotely, 446 (23%) only recently, and 299 (15%) only remotely. Median length of stay was 13 days (IQR 6–28).

A total of 142 cases (7%) did not have hospitalization or outpatient AU in the prior year, and 1,097 (56%) had both.

**Conclusion:**

Incident CDI cases have substantial exposure to recent and remote hospitalization and outpatient AU. Understanding cumulative effects of multiple risk factors can guide prevention strategies, including antibiotic stewardship efforts.

**Disclosures:**

**Scott Fridkin, MD**, Pfizer: Grant/Research Support **Ghinwa Dumyati, MD**, Pfizer: Grant/Research Support.

